# A Semisupervised Majority Weighted Vote Antiphishing Attacks IDS for the Education Industry

**DOI:** 10.1155/2022/7402085

**Published:** 2022-03-31

**Authors:** Xiaona Yin, Xingxing Zheng

**Affiliations:** Zhengzhou Preschool Education College, Zhengzhou 450000, China

## Abstract

Although the digital transformation is advancing, a significant portion of the population in all countries of the world is not familiar with the technological means that allow malicious users to deceive them and gain great financial benefits using phishing techniques. Phishing is an act of deception of Internet users. The perpetrator pretends to be a credible entity, abusing the lack of protection provided by electronic tools and the ignorance of the victim (user) to illegally obtain personal information, such as bank account codes and sensitive private data. One of the most common targets for digital phishing attacks is the education sector, as distance learning became necessary for billions of students worldwide during the pandemic. Many educational institutions were forced to transition to the digital environment with minimal or no preparation. This paper presents a semisupervised majority-weighted vote system for detecting phishing attacks in a unique case study for the education sector. A realistic majority weighted vote scheme is used to optimize learning ability in selecting the most appropriate classifier, which proves to be exceptionally reliable in complex decision-making environments. In particular, the voting naive Bayes positive algorithm is presented, which offers an innovative approach to the probabilistic part-supervised learning process, which accurately predicts the class of test snapshots using prerated training snapshots only from the positive class examples.

## 1. Introduction

The consequent increase in the popularity of online educational resources, combined with the lack of preparedness, has made the education sector an ideal target for digital phishing attacks [1]. Phishing is the most widespread technique where malicious users create fake websites that look like the official websites of legal organizations/companies/banks [2, 3]. They then send emails or SMS or create misleading messages that link to the misleading URL they have made. Users are asked to fill in confidential personal and financial data on these websites, including usernames, passwords, and bank card details. The main reasons cited by most phishing messages are a problem in the user's account, a confirmation of execution or cancellation of a transaction (which has never been done by the user), a service upgrade action, and so on [4].

A successful phishing attack is based on the victim's lack of knowledge, attention, and visual deception [3]. The average person knows how to handle the essential functions of the computer and the Internet without knowing the process by which it works. So, it cannot recognize traces of phishing, such as a varied e-mail address or a different URL. At the same time, due to ignorance of the risk, the user neglects antiphishing programs. Even in cases where the users have the appropriate knowledge to detect malicious elements, they often will not notice the signs, as they may be abstract or busy with something else. Thus, the user may not pay enough attention to the current security warnings or lack them. After all, the proper phishing technique hides most signs as a successful phishing attack is based mainly on visual deception. The aim is to convince the victim of the authenticity and reliability of the fraud, which is achieved by [5, 6] the following.

1. *Misleading Text.* This text, which is usually misleading links, may use incorrect syntax or spelling, for example, www.fasebook.com, anagrams, e.g., and www.yutoube.com, or replace similar letters such as the English lowercase *l* (*L*) with the capital I (i).
2. *Misleading Images.* These images may be visually the same as the images used by a website, for example, the Google logo, but when you click on them, they redirect you elsewhere. An equally standard method is images that mimic the computer operating system.
3. *Misleading Design.* With the help of misleading text and images and the processing of the code of the original website, the malicious user can create an entire website with the same design as the original.
4. *Threatening Message.* The message usually contains a threat or a problem that the user must deal with. For example, “if you do not follow the link, your account will be locked,” or “as soon as a transaction was made from your account, click here to cancel it.”

If a phishing campaign manages to combine all the above, it will be successful in most cases. The research community intensively deals with this cyber threat, while many of their research results have been presented in the international literature [6–10].

Section 2 includes an overview of approaches identified in the literature and associated with similar technical standardization. You will discover more about the suggested system's technique in Section 3. According to the dataset and findings presented in Section 4 of the proposed approach, there are no restrictions on applying it.  Section 5 finishes with a summary of the findings and a list of possible next research directions.

## 2. Literature Review

The concept of phishing attack detection has been approached with various methods from the research community. During the last five years, especially, researchers have been evaluating machine learning approaches to face this rising problem better.

Cuzzocrea et al. [4] offered a machine learning-based approach for detecting the difference among phishing and authentic websites. They built signs to identify phishing activity using cutting-edge machine learning techniques. The suggested solution is based on a simple feature vector to collect and does not need extra processing. They stated that by evaluating a certain algorithm, they might get encouraging results in identifying phishing attempts.

Natural language processing methods were utilized by Peng et al. [11] to evaluate text (but not message metadata) and identify incorrect utterances indicative of phishing attempts. To identify harmful information, they used a semantic analysis of the text. Their strategy resulted in entirely text-based phishing emails, with no harmful attachments attached. They tested it with a huge batch of phishing emails and found that it had a high recall rate, proving that semantic information is a good predictor of social engineering.

Garces et al. [6] conducted a study on examining anomalous behavior associated with phishing online assaults and how machine learning methods may be used to combat the issue. This assessment was done using infected data sets and scripting language tools to establish machine learning for detecting phishing attacks throughout the analysis of URLs to determine if they were good or bad URLs based on specific characteristics of the URLs and to provide real-time information and making informed decisions that reduce the potential damage.

Basit et al. [2] conducted a study of Artificial Intelligence approaches in use, including spoofing attack mitigations tactics, data mining and heuristics, machine learning, and AI techniques. They also evaluated several research for each AI technology that detected phishing attacks and looked at the benefits and drawbacks of each methodology. Compared with other classification techniques such as random forest, support vector machine, decision tree, principal component analysis, and k-nearest neighbor, Machine learning processes provide the most significant results. Future study towards a more configurable strategy, including creative plugin solutions to tag or label whether a website is genuine or leading to a phishing attempt, is suggested.

Saha et al. [5] established a data-driven approach utilizing a feed-forward neural network to anticipate phishing websites. Their program was able to classify websites into three categories: phishing, suspicious, and authentic. The dataset was large, including data from hundreds of web pages, and their model had excellent training and test accuracy percentages. The difference between training and test accuracy was small, indicating that the proposed model learned from the dataset and was capable of quickly detecting unfamiliar web pages. The authentic website identification accuracy, on the other hand, was greater than the existing phishing detection method.

Using machine learning methods such as random forest and decision tree, Alam et al. [7] created a model to identify phishing assaults. To detect phishing, the study used a variety of tactics. The machine learning algorithms were fed standard datasets of phishing assaults from kaggle.com. The suggested model uses feature selection methods like principal component analysis to identify and categorize the datasets' components to study their properties. To categorize the website, a decision tree was employed, and random forest was used for categorization. Finally, a confusion matrix was created to compare the two algorithms' efficiency. The random forest algorithm has a 97 percent accuracy rate. The study team intends to use a convolution neural network to anticipate phishing attempts from a recorded dataset of attacks, which might be included as a tool for intrusion detection systems.

Finally, Singh et al. [12] conducted a survey where they compared 16 distinct study studies. Network-level security, authentication, client-side tools, server-side filters, and user education were the three classes they used to categorize phishing defenses. They came to the conclusion that the research community is still unable to give a “silver bullet” for spoofing attack defense.

As many schools and universities conduct classes online, these organizations must take steps to secure their digital learning environments [13, 14]. The proposed approach of the work aims to detect malicious URLs related to phishing attacks, to predict vulnerabilities, which may come from fraud or cyber-attacks.

## 3. Proposed Methodology

The primary idea of the proposed methodology is based on an algorithmic approach of the naive Bayes positive classifier [15]. This offers a simple probabilistic approach to part-supervised learning problems. Our goal is to accurately predict the class instance of instantaneous instruction only from the positive class and several unsorted examples. The probabilities that we have to calculate, using only the positive and unclassified examples that we have at our disposal, are the ex-ante probabilities of observing positive and negative examples *p*(*C*=pos) and *p*(*C*=neg), respectively, as well as the ex-ante probabilities of occurrence of each attribute, for each class (i.e., *p*(*X*_*ι*_=*x*_*i*_*|C*=*pos*) and *p*(*X*_*ι*_=*x*_*i*_*|C*=neg). Due to the absence of negative examples, it is impossible to define the *p*(*C*=pos), so the user must give an approximation. Let $\hat{p}\left(pos\right)$, so that *p*(*C*=neg) is calculated as follows [16]:

(1) $$\begin{array}{c} p\left(C=\text{neg}\right)=1-\hat{p}\left(\text{pos}\right). \end{array}$$

In terms of the probabilities of the features given a positive class, *p*(*X*_*ι*_=*x*_*i*_*|C*=pos)it is estimated strictly for the different types of components [17, 18]:

(2) $$\begin{array}{c} p\left(X_{i}=x_{i}|C=c\right)=g\left(x_{i};\mu_{i,c},\sigma_{i,c}\right), \end{array}$$

while for the estimation of *p*(*X*_*ι*_=*x*_*i*_*|C*=neg), we use the law of total probability [16, 19]:

(3) $$\begin{array}{c} p\left(X_{i}=x_{i}\right)=p\left(X_{i}=x_{i}|C=\text{pos}\right)p\left(C=\text{pos}\right)+p\left(X_{i}=x_{i}|C=\text{neg}\right)p\left(C=\text{neg}\right)\Rightarrow \\ p\left(X_{i}=x_{i}|C=\text{neg}\right)=\frac{p\left(X_{i}=x_{i}\right)-p\left(X_{i}=x_{i}|C=\text{pos}\right)p\left(C=\text{pos}\right)}{1-p\left(C=\text{pos}\right)}, \end{array}$$

where everything is known except the ex-ante probability of occurrence of the characteristic *X*_*ι*_, *p*(*X*_*ι*_=*x*_*i*_), which is approximated by assuming that the set UD of the unsorted examples follows the distribution of real-world examples.

The *p*(*X*_*ι*_=*x*_*i*_*|C*=neg) approach runs the risk of being negative. Therefore, we need to replace the negative values with 0 and normalize our practices, so that they all have a sum of 1. This is a simple case for the discrete attributes since the domain definition of the attribute takes discrete values, making it possible to calculate them all to normalize them. But, for continuous features, we create a new distribution (normal distribution or sum of Gaussian nuclei). Under the previously mentioned conditions (assumptions), the proposed algorithm that we use in this work is as follows [15, 20–22].

Let us assume a data training body with only positive PD examples and a body of unclassified UD data. Also, let $\hat{p}\left(pos\right)$ estimate the ex-ante probability of the positive class. The naive Bayes positive classifier classifies an unknown *x* instance as a member of the class [15, 19]:

(4) $$\begin{array}{c} \underset{ce\left\{\text{pos,neg}\right\}}{\arg\text{max}}\left\{p\left(C=c|X=x\right)\right\}=\underset{c\in \left\{\text{pas,neg}\right\}}{\arg\text{max}}\left\{p\left(C=c\right)\prod p\left(X_{i}=x_{i}|C=c\right)\right\}. \end{array}$$

The estimates of the ex-ante probabilities of the classes are calculated from

(5) $$\begin{array}{c} p\left(C=\text{pos}\right)=\hat{p}\left(\text{pos}\right),p\left(C=\text{neg}\right)=1-\hat{p}\left(\text{pos}\right). \end{array}$$

The estimates of the likelihood of the features are calculated for the discrete elements:

(6) $$\begin{array}{c} p\left(X_{i}=x_{i}|C=\text{pos}\right)=\frac{\#\left(x_{i},\text{PD}\right)}{\left|P\;D\right|}\\ p\left(X_{i}=x_{i}\right)=\frac{\#\left(x_{i},\text{UD}\right)}{\left|U\;D\right|}. \end{array}$$

For continuous features using Gaussian distribution [23, 24],

(7) $$\begin{array}{c} p\left({X_{i}=x|}_{i}C=\text{pos}\right)=g\left(x_{i};\mu_{i,c},\sigma_{i,c}\right)p\left(X_{i}=x_{i}\right)=g\left(x_{i};\mu_{i},\sigma_{i}\right). \end{array}$$

For continuous features using Gaussian kernels,

(8) $$\begin{array}{c} p\left(X_{i}=x_{i}|C=\text{pos}\right)=\frac{1}{\left|\text{PD}\right|}\sum_{j}g\left(x_{i};{\left(x_{i}\right)}_{j},\frac{1}{\sqrt{\left|P\;D\right|}}\right),\\ p\left(X_{i}=x_{i}\right)=\frac{1}{\left|\text{UD}\right|}\sum_{J}g\left(x_{i};{\left(x_{i}\right)}_{j},\frac{1}{\sqrt{\left|U\;D\right|}}\right). \end{array}$$

For all the previously mentioned cases, the following applies:

(9) $$\begin{array}{c} p\left(X_{i}=x_{i}|C=\text{neg}\right)=\frac{p\left(X_{i}=x_{i}\right)-p\left(X_{i}=x_{i}|C=\text{pos}\right)p\left(C=\text{pos}\right)}{1-p\left(C=pos\right)}, \end{array}$$

which is normalized so that

(10) $$\begin{array}{c} p\left(X_{i}=x_{i}|C=\text{neg}\right)=\text{max}\left\{p\left(X_{i}=x_{i}|C=\text{neg}\right);0\right\}\text{ and }\sum_{\forall x}p\left(X_{i}=x|C=\text{neg}\right)=1, \end{array}$$

where *x* takes values from the definition field of *X*_*i*_.

Given that PD is the set of positively sorted examples and UD is the set of nonsorted, a first not satisfying approach is to assume that all unknown models are negative, so

(11) $$\begin{array}{c} \hat{p}\left(\text{pos}\right)=\frac{\left|\text{PD}\right|}{\left|\text{PD}\right|+\left|\text{UD}\right|}. \end{array}$$

But since there will also be positive examples in the unclassified UDs, a better approach to $\hat{p}\left(pos\right)$ would be to add the number of these positive examples to the numerator of the above fraction. We construct the first classifier to classify the unknown samples using the simple hypothesis that all unknowns are negative. The number of positive examples to be found is added to the numerator of the above fraction, a new approximation of $\hat{p}\left(\text{pos}\right)$ is calculated, and a new classifier is constructed to reclassify the unknown examples [15, 19]:

(12) $$\begin{array}{c} \hat{p}\left(\text{pos}\right)=\frac{\left|\text{PD}\right|+\left|\text{most\_probable\_positive\_from}\left(\text{UD}\right)\right|}{\left|\text{PD}\right|+\left|\text{UD}\right|}. \end{array}$$

This process is repeated until $\hat{p}\left(pos\right)$ converges, remaining the same in two consecutive steps. However, because not every single classifier can be optimal for all metrics, we will use a voting scheme, that is, a combination of classifiers, to derive the optimal characteristics for all performance metrics as a decision rule based on the predicted class with the most votes.

Specifically, because we have at least two independent, equivalent classifiers which make a single decision on the class of the unlabeled sample, this sample is classified in the class where there is an absolute majority, that is, a decision agreed by at least half of the experts. To make the system more realistic, the decision of each classifier is multiplied by a weight that reflects the individual confidence in its conclusions. The more reliable the classifier is in its choices, the higher the weight value assigned to it. The sum of the weights is equal to one. Therefore, if the decision of the *k* classifier to classify the unknown sample in the *i* class is given by *d*_*ik*_ with 0 ≤ *i* ≤ *m*, where *m* is the number of classes, then the final combined decision for assignment to class *I* is as follows [25, 26]:

(13) $$\begin{array}{c} d_{i}^{\text{com }}=\sum_{i=1,2,\ldots ,m}\omega_{k}*d_{ik}. \end{array}$$

Therefore, the class *y* is the one selected if *d*_*y*_^com ^ is the maximum. To find the optimal values of the weights, they must minimize the error function defined as

(14) $$\begin{array}{c} y\neq \text{true\_label for max}\left(d_{y}^{\text{com}}\right). \end{array}$$

A decision function is optimal when the previously mentioned formula is minimized in all possible decisions. Assuming independence between classifiers and that if the probability of selecting class *i* is *p*_*i,*_ then the likelihood of choosing any other class is evenly distributed among them, we arrive at a majority weighted vote approach [17, 19, 20].

(15) $$\begin{array}{c} f^{\text{opt}}\left(x\right)=\text{sign}\left(\sum_{i=1}^{n}\omega_{i}*x_{i}\right). \end{array}$$

The weights *ω*_*i*_ are given by the relation:

(16) $$\begin{array}{c} \omega_{i}=\text{log}\left(\frac{p_{i}}{1-p_{i}}\right),\;i\in \left[n\right], \end{array}$$

where *p*_*i*_ is the probability that the specialist will choose class *i*.

The calculation of the weights by approaching the joint probability distribution for each class with a set of answers of the classifiers is as follows:

(17) $$\begin{array}{c} \text{P}\left(c|f_{1},\ldots ,f_{v}\right)=\frac{p\left(c\right)*P\left(f_{1},\ldots ,f_{v}|c\right)}{P\left(f_{1},\ldots ,f_{v}\right)}, \end{array}$$

where *f*_1_ is the attribute, and *c* is the variable for the class. Assuming independence between the features we have from the previous formula

(18) $$\begin{array}{c} P\left(c|f_{1},\ldots ,f_{v}\right)=\frac{1}{Z}p\left(c\right)*\prod_{i=1}^{v}p\left(f_{i}|c\right). \end{array}$$

We observe that *Z* is a multiplication factor and is independent of the variable class *c*. Taking as random variables all the answers of the classifiers instead of the characteristics, we end up with the following:

(19) $$\begin{array}{c} \text{P}\left(c|e_{1},\ldots ,e_{k}\right)=\frac{1}{Z}p\left(c\right)*\prod_{i=1}^{k}p\left(e_{i}|c\right). \end{array}$$

Given the relation,

(20) $$\begin{array}{c} P\left(c,e_{1},\ldots ,e_{k}\right)=P\left(c|e_{1},\ldots ,e_{k}\right)*Z, \end{array}$$

that is, replacing the bound probability with the common ones, we conclude from the previous formula [19, 24]:

(21) $$\begin{array}{c} P\left(c,e_{1},\ldots ,e_{k}\right)=p\left(c\right)*\prod_{i=1}^{k}p\left(e_{i}|c\right). \end{array}$$

Therefore, the weights are related to the variable of class *u* with the relation:

(22) $$\begin{array}{c} \omega \left(e_{1},\ldots ,e_{k}\right)=p\left(c=u\right)*\prod_{i=1}^{k}p\left(e_{i}|c=u\right). \end{array}$$

Thus, the class $\hat{c}$ of the unlabeled sample *x* is calculated as

(23) $$\begin{array}{c} \hat{c}=\underset{u\in C}{\text{max}}\sum_{i}\omega_{u}*r_{i,u}. \end{array}$$

Therefore, given each input sample *x* and set of answers of the classifiers, the weights are calculated, and the final decision is made based on the equation of $\hat{c}$.

**Figure 1:**
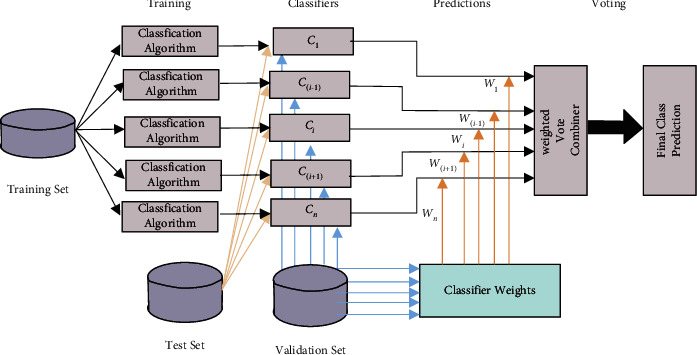
The majority weighted vote methodology.

A depiction of the proposed methodology is presented in Figure 1.

## 4. Dataset and Results

In the present study, we used data from the PhishTank database, a complete database for registrations for Phishing URLs. A total of 860,000 URLs were used, of which 500,000 were legit, and 360,000 were phishing. The export of features was based on the idea that URLs are divided into subsections as explicitly shown in domain, directory, file, and parameters. In each section, we measure the number of some special characters (e.g., -, #, @, etc.) and the size of the section and check if certain words appear in specific sections (e.g., “client,” “server,” “script,” etc.) and if there is an IP or e-mail in the domain section, as well as the number of vowels in the domain. In addition, there are features based on external services (WHOIS2, HTTPS3 Protocol, SSL4 certificate, etc.) and components based on the number of occurrences of specific HTTP headers (e.g., cookies; strict-transport-security). The following features were extracted in detail from each URL:

1. check_ssl: check for valid SSL protocol (0 False - 1 True)
2. url_redirect: Number of redirects (numeric value)
3. url_shortened: URL shortcut control (0 False - 1 True)
4. favicon: check if the favicon is loaded from an external domain (0 False - 1 True)
5. dns_record: check for DNS domain registration in WHOIS (0 True - 1 False)
6. iFrame: iFrame existence check (0 False - 1 True)
7. rightClick: check if right-click is disabled (0 True - 1 False)
8. onmouseover: check if onmouseover changes the status bar (0 True - 1 False)
9. check_URL_anchor: check if anchors lead to a new domain (real percentage)
10. sfh: check if the action of a form tag triggers an action (0 False - 1 True)
11. double_slash: Existence “//” more than 1 time in the URL (0 False - 1 True)
12. url_dot_url: Number of “.” in full URL (numeric value)
13. url_hyphen_url: Number of “-” in the whole URL (numeric value)
14. url_questionmark_url: Number of “?” in full URL (numeric value)
15. url_at_url: Number of “@” in the whole URL (numeric value)
16. url_hashtag_url: Number of “#” in the whole URL (numeric value)
17. url_dollar_url: Number of “$” in the whole URL (numeric value)
18. url_percent_url: Number of “%” in the whole URL (numeric value)
19. tld_length: Number of TLD5 (numeric value)
20. tld_count: Number of sub-TLDs (numeric value)
21. url_length: Number of characters in the entire URL (numeric value)
22. e-mail_in_url: Show e-mail inside URL (0 False - 1 True)
23. word_script_in_url: Display the word “script” inside the URL (0 False - 1 True)
24. check_https_in_url: Display the word “https” inside the URL (0 False - 1 True)
25. url_dot_domain: Number of “.” in the Domain section (numeric value)
26. url_hyphen_domain: Number of “-” in the Domain section (numeric value)
27. count_vowels: Number of vowels in the Domain section (numeric value)
28. domain_length: Number of characters in the Domain section (numeric value)
29. ip_in_domain: Display IP in the Domain section (0 False - 1 True)
30. client_or_server_domain: Display client or server in Domain (0 False - 1 True)
31. check_age_of_domain: WHOIS Domain Registration Days (numeric value)
32. days_till_expiration_domain: Days until SSL expires (numeric value)
33. url_dot_directory: Number of “.” in the Directory section (numeric value)
34. url_hyphen_directory: Number of “-” in the Directory section (numeric value)
35. url_at_directory: Number of “@” in the Directory section (numeric value)
36. url_slash_directory: Number of “/” in the Directory section (numeric value)
37. url_percent_directory: Number of “%” in the Directory section (numeric value)
38. directory_length: Length of in the Directory section (numeric value)
39. url_dot_File: Number of “.” in the File section (numeric value)
40. url_hyphen_File: Number of “-” in File section (numeric value)
41. url_at_File: Number of “@” in the File section (numeric value)
42. url_percent_File: Number of “%” in File section (numeric value)
43. file_length: Number of characters in the File section (numeric value)
44. url_dot_params: Number of “.” in the Params section (numeric value)
45. url_hyphen_params: Number of “-” in the Params section (numeric value)
46. url_at_params: Number of “@” in the Params section (numeric value)
47. url_underline_params: Number of “_” in Params section (numeric value)
48. url_hashtag_params: Number of “#” in the Params section (numeric value)
49. url_dollar_params: Number of “$” in the Params section (numeric value)
50. url_percent_params: Number of “%” in the Params section (numeric value)
51. params_length: Number of characters in the Params section (numeric value)
52. tld_params: check if there are any of the TLDs in Params (0 False - 1 True)
53. count_params: Number of parameters to get a value (numeric value)
54. cookie: check if the HTTP header adds a cookie (0 False - 1 True)
55. strict_trans_sec: check for HTTP header to switch to HTTPS (0 False - 1 True)
56. a_tags_count: Number of tags in the HTML code of the web page (numeric value)
57. form_tags_count: Number of form tags in HTML code (numeric value)
58. e-mail_tags_count: Number of “emails” displayed in HTML code (numeric value)
59. pass_tags_count: Number of “password” occurrences in HTML code (numeric)
60. hidden_tags_count: Number of hidden tags in HTML code (numeric value)
61. actions_tags_count: Number of action tags in HTML code (numeric value)
62. signin_tags_count: Number of “sign in” occurrences in HTML code (numeric)
63. signup_tags_count: Number of “sign up” occurrences in HTML code (numeric)
64. label: for the type of URL (0 legitimate - 1 phishing)

To prove the possibility of the proposed scheme, we made a comparison with known machine learning methods. The results of the process are presented in Table 1.

Although all the models achieve high success rates, the proposed one achieved the highest success rates. With the voting naive Bayes positive technique [15, 19] that we propose, we perform the highest percentages for accuracy, precision, recall, and F1, which indicates the possibility of generalization of the proposed system. Also, the metric MCC, which is used as a measure of the quality of the categorization, and the high results of the proposed method prove that the coefficient considers the TP, FP, TN, and FN, which ensures a very balanced performance in cases where the two classes have different sizes, as in the problem that concerns us. The MCC is essentially a correlation coefficient between the predicted and observed values of the categorization, and it takes values between -1 and +1. A factor of +1 represents a perfect prediction. If its value is 0, the categorizer prediction is no better than a random prediction. When its value is -1, there is a total difference between the forecast price and the real one. While there is no perfect way to describe the results of a single numbered confusion matrix, the metric MCC is considered one of the best. The methodology in question also strengthened the weighting process in the majority weighted vote process and how the model weightings were calculated [27, 28].

**Figure 2:**
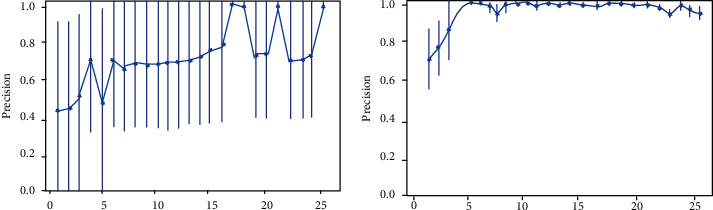
Precision majority vote (left) vs. precision weighted vote (right).

Also, the majority weighted vote process leads to better performance of the final model because it reduces model variability without significantly increasing bias. This means that while the predictions of an individual model are pretty sensitive to the noise of the training set, the weighted average of the results of many classifiers is not if they are not correlated with each other. This happens here due to the method followed since different classifiers see different points of the education set. A typical example of proof of this fact is in Figure 2, which clearly shows the performance of the classifiers with the two different procedures and the apparent superiority of the proposed majority weighted vote.

In general, with the majority weighted vote procedure followed, even if the relative majority agrees with the prevalence of a class, the uncertainty about their prediction against the firm opinion of the two models would lead to a wrong result by a majority vote. On the other hand, although theoretically ensuring significant percentages in the evaluation metrics and showing commendably good results, a simple voting process does not consider the general cases of class inhomogeneity, so the forecasts do not guarantee a final result based on generalization.

In conclusion, the operation and the results of the application are considered very satisfactory, which should also be noted that it manages to detect phishing websites from the first minute they are published, in contrast to the browsers and databases of cybersecurity companies, which require some time-space, maybe a lot of reports from users.

## 5. Conclusions

The consequent increase in the popularity of online educational resources, combined with the lack of preparedness, has made the education sector an ideal target for digital phishing attacks. The identification and timely assessment of these threats to the functioning of educational organizations allow the detection of incidents and the corresponding identification of correlations and causal relationships with security incidents, which can significantly mitigate the effects of organized cyber attacks. In this spirit, a semisupervised majority-weighted voting system for detecting phishing attacks was proposed in this paper. Specifically, the voting naive Bayes positive algorithm was used, which offers an innovative approach to the probabilistic learning process with partial supervision. Our goal is to accurately predict the class-class of test snapshots using both classified and positive training snapshots, as well as a variety of unclassified examples.

This algorithmic process, which we presented for the first time in the literature, was evaluated in a very complex problem of identifying URLs related to phishing attacks in a timely scenario associated with the educational process. A very complex but ideal dataset was used, which computes the problem of phishing attacks in the educational sector in a complete way, and the proposed algorithm achieved very high generalization rates.

Future research for the extension of the proposed system is related to implementing the system with more classes to reveal in more detail the system's ability to model more complex problems. It would also be essential to identify ways the system can receive information from a posteriori or a priori probabilities in a complete predictive environment with retrospective relationships. For example, the method by Bayesian inference will be enhanced, which is a method of statistical inference, where Bayes' theorem is used to update the probability for a hypothesis as more evidence or information becomes available.

## Figures and Tables

**Table 1:** Performance measures.

Model	Accuracy	Auc	Recall	Prec.	F1	Kappa	MCC	TT (sec)
Voting naive bayes positive	0.9314	0.9982	0.9292	0.9320	0.9312	0.8722	0.8871	2.339
Light gradient boosting machine	0.8949	0.9777	0.8770	0.8970	0.8941	0.8197	0.8218	0.244
Extreme gradient boosting	0.8942	0.9759	0.8745	0.8976	0.8935	0.8187	0.8211	15.896
CatBoost classifier	0.8926	0.9763	0.8710	0.8950	0.8921	0.8154	0.8172	4.328
Random forest classifier	0.8918	0.9739	0.8685	0.8961	0.8918	0.8145	0.8169	0.562
Gradient boosting classifier	0.8864	0.9747	0.8635	0.8914	0.8861	0.8053	0.8082	0.665
SVM - radial kernel	0.8726	0.9498	0.8388	0.8765	0.8716	0.7806	0.7832	0.387
k-Neighbors classifier	0.8687	0.9494	0.8336	0.8700	0.8666	0.7727	0.7753	0.128
MLP classifier	0.7988	0.8728	0.8076	0.7877	0.7541	0.7719	0.7056	6.322

## Data Availability

Data are available on reasonable request.
